# Comparative analysis of the transcriptomes of the calyx abscission zone of sweet orange insights into the huanglongbing-associated fruit abscission

**DOI:** 10.1038/s41438-019-0152-4

**Published:** 2019-06-01

**Authors:** Wei Zhao, Elizabeth A. Baldwin, Jinhe Bai, Anne Plotto, Mike Irey

**Affiliations:** 1 USDA/ARS Horticultural Research Laboratory, 2001 South Rock Road, Fort Pierce, FL 34945 USA; 2 Southern Gardens Citrus Nursery, 111 Ponce de Leon Avenue, Clewiston, FL 33440 USA

**Keywords:** Transcriptomics, Biotic

## Abstract

Citrus greening disease or huanglongbing (HLB) is associated with excessive pre-harvest fruit drop. To understand the mechanisms of the HLB-associated fruit abscission, transcriptomes were analyzed by RNA sequencing of calyx abscission zones (AZ-C) of dropped “Hamlin” oranges from HLB-diseased trees upon shaking the trees (Dd), retained oranges on diseased trees (Rd), dropped oranges from healthy shaken trees (Dh), and retained oranges on healthy trees (Rh). Cluster analysis of transcripts indicated that Dd had the largest distances from all other groups. Comparisons of transcriptomes revealed 1047, 1599, 813, and 764 differentially expressed genes (DEGs) between Dd/Rd, Dd/Dh, Dh/Rh, and Rd/Rh. The gene ontology (GO) and Kyoto Encyclopedia of Genes and Genomes (KEGG) pathway enrichment analyses indicated hormone signaling, defense response, and secondary metabolism were involved in HLB-associated fruit abscission. Ethylene (ET) and jasmonic acid (JA) synthesis/signaling-related genes were upregulated in Dd, while other phytohormone-related genes were generally downregulated. In addition, genes related to JA/ET-activated defense response were upregulated in Dd as well. Consistent with the phytohormone gene expression data, increased levels (*p* < 0.05) of ET and JA, and a decreased level (*p* < 0.05) of abscisic acid were found in Dd compared with Rd, Dh or Rh. *Lasiodiploidia theobromae* level in Dd AZ-C was higher than the other fruit types, confirmed by qPCR, indicating AZ-C secondary fungal infection of HLB fruit may exacerbate their abscission. This information will help formulate effective strategies to control HLB-related abscission.

## Introduction

The shedding or abscission of citrus-reproductive organs is a highly regulated process that is influenced and activated in response to exogenous (changing environmental conditions, interactions with microorganisms) and endogenous (physiological modifications) stimuli. The process takes place at anatomically distinct cell layers termed abscission zones (AZ)^1^. Bud and flower abscission takes place at the abscission zone A (AZ-A) located between twig and peduncle, whereas fruitlet and fruit abscission occurs at the AZ located at the calyx between the pericarp and the floral disc (calyx abscission zone or AZ-C)^2^. AZ comprises distinct layers of small cytoplasmic dense cells that forms at an early stage of pedicel development and proliferates during fruit development^3^. Major events leading to abscission have been classified into three phases to occur: differentiation and development of the AZ tissue, acquisition of competence to respond to abscission signals, and execution (activation) of abscission following perception of abscission signals^3^. Over the past decade, molecular studies in model plants (tomato and Arabidopsis) have identified key molecular components involved in the development of AZ or regulation of abscission^4–12^. Three members of MADS-box family (J, MC, and SlMBP21) have been found to interactively play a central role in pedicel AZ development^4–7^. Ethylene-response factor (ERF) family gene (*SlERF52*) has been identified as a connector between abscission-inducing signals and abscission processes^8^, and the factors that regulate AZ activities (transcription factors KNOX and bHLH; leucine-rich repeat (LRR) receptor-like kinases, small signaling peptide IDA) have been also reported^9–12^. In citrus, transcriptome analyses identified genes regulated in AZ-C during ethylene-promoted abscission, including activation of ethylene-responsive transcription factors, genes involved in defense, cell wall degradation and secondary metabolism, and downregulation of genes involved in starch/sugar biosynthesis and growth-promoting hormone synthesis^13,14^.

The responses of AZ cells to internal and external abscission-triggering signals are mediated by phytohormones^15^. Ethylene (ET), jasmonic acid (JA), and abscisic acid (ABA) are abscission accelerators, whereas auxins, gibberellins, and brassinosteroids inhibit abscission^15,16^. With the recent recognition of JA’s promotion roles in abscission, the interactions of JA and ET have been revealed and the interplay between JA and ET was found to play a role in AZ’s acquisition of competence for activation of abscission^17,18^.

Although abscission of reproductive organs is somewhat continuous during citrus fruit setting, growth, and ripening, two waves of elevated abscission of the ovary or young fruit take place after anthesis^2^. The first occurs after anthesis, due to poor or lack of pollination^2^, or caused by the fungus *Colletotrichum acutatum* which occurs in humid citrus production areas^19^; and the second one usually occurs about 1-month later (referred to as “June drop”), mainly due to insufficient supply of carbohydrates to young fruit^20^. When the fruit approach maturity, fruit abscission is considerably reduced, and non‐climacteric citrus fruit lack a well-defined abscission period. Fruit can hang on trees for a few months to a half-year, depending on cultivars and environmental and physiological conditions^2^. However, in the case of citrus greening or huanglongbing (HLB)-affected citrus, there is excessive pre-harvest fruit abscission as the fruit reaches final maturity, and this causes a reduction in yield^21^.

HLB is a devastating disease of citrus putatively caused by the phloem-limited proteobacterium *Candidatus* Liberibacter asiaticus (*C*Las). Typical symptoms of HLB-diseased trees include leaf chlorosis (“blotchy mottle”), twig dieback, and canopy thinning. Fruit symptoms often manifest as reduced fruit size, misshapen fruit, and poorly colored with an inverted color change (coloration beginning at the stem end). An orange–brown stain may be present in the AZ-C^22^. Many fruit abscise prematurely^21^.

The mechanism of HLB-associated excessive pre-harvest fruit abscission has remained undetermined. Two research groups have recently studied mature citrus fruit abscission in response to ET treatment by analyzing global gene expression in ET-promoted AZ-C of citrus fruit^13,14^; however, no HLB-affected fruit were included in these studies. Since excessive accumulation of starch, callose depositions, and phloem plugging in leaves and stems, and a loss of fibrous roots have been found in HLB-affected citrus trees^23^, it has been proposed that the water, carbohydrate, or nutrient shortage due to the root loss/phloem plugging may be responsible for the excessive pre-harvest fruit drop^24^. This hypothesis, however, still lacks evidence. The enhanced nutritional programs adopted by growers to reduce tree disease symptoms, unfortunately, did not affect fruit drop^25^; while the attempts to increase root density to alleviate the fruit drop problem have not been proven to be significantly effective^26^. Other recent studies linked fungus *Lasiodiplodia theobromae* (Diplodia) infection of the fruit AZ-C to an increase in fruit ET production and a decrease in fruit detachment force^27,28^. Spraying trees with fungicide during the season reduced Diplodia infection and fruit drop for some varieties^28^.

In order to provide a comprehensive understanding of HLB-associated fruit abscission at the molecular level, in this study, we conducted comparative transcriptomics analysis of the AZ-C between the “Hamlin” oranges that were loosely and tightly held to the HLB-diseased trees, and compared with those from healthy trees. Four types of fruits were used in the study: fruit that dropped from the HLB-diseased trees upon shaking the trees (Dd), fruit that remained on the HLB-diseased trees (subsequently harvested for the study) when shaking the trees (Rd), fruit that dropped from healthy trees when vigorously shaking the trees (Dh), and the fruit that remained on the healthy trees (subsequently harvested) when shaking the trees (Rh). Genes, pathways, and biological processes that are involved in HLB-associated pre-harvest fruit drop were identified by genome-wide gene expression profiling and enrichment analyses. The information will facilitate formulation of an effective strategy to control HLB-related fruit drop.

## Results

### Transcriptome profiling and identifying differentially expressed genes (DEGs)

**Fig. 1:**
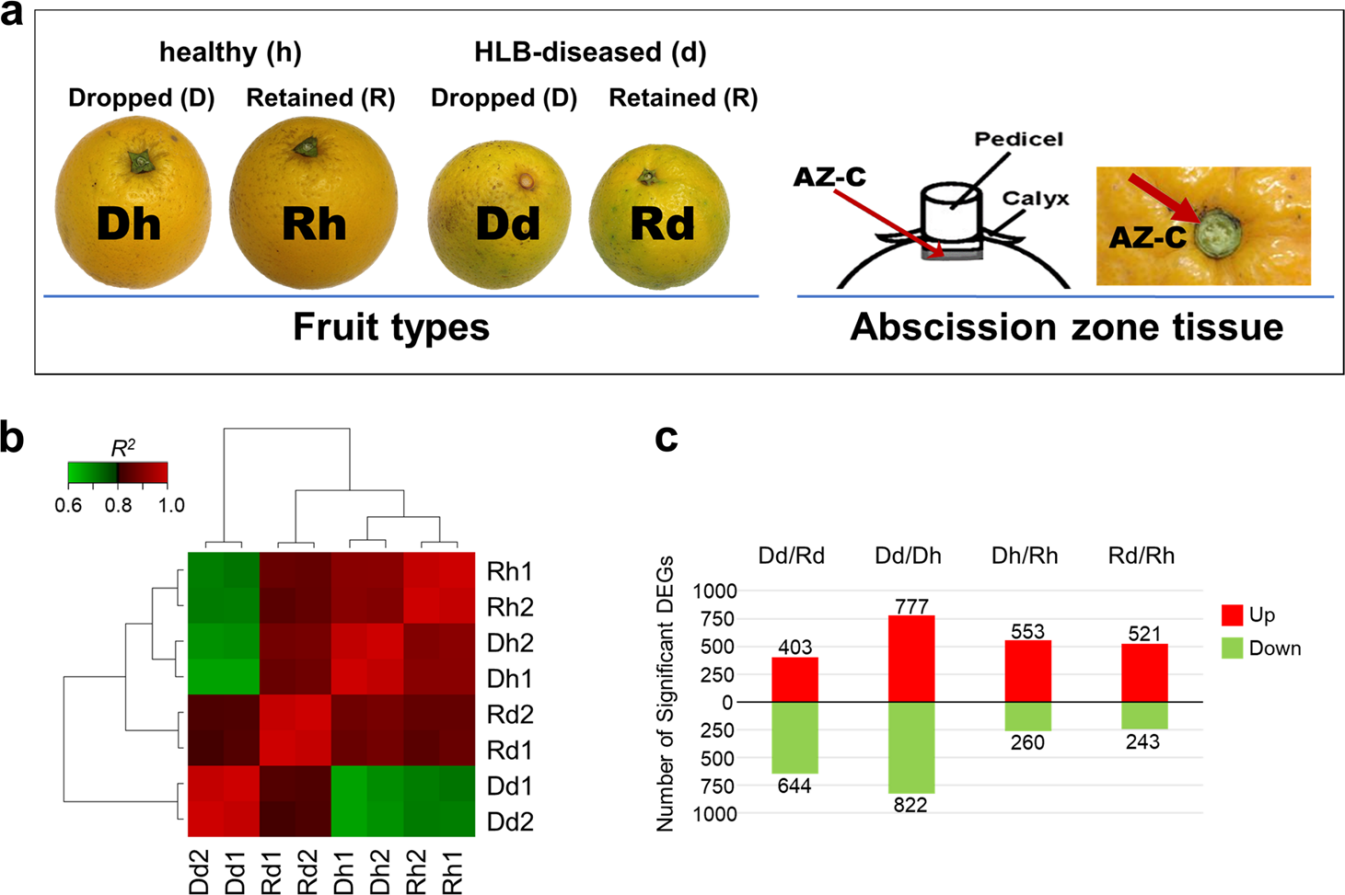
Images/Diagrams illustrating the fruit calyx abscission zone (AZ-C) tissue, correlations/distances among transcriptomes and the number of differentially expressed genes in AZ-C of different types of fruit. a The fruit types and abscission zone tissue used in the experiment. **b** Correlation matrix and cluster dendrogram of the whole dataset of the mapped reads. The analysis was performed by comparing the values of the entire transcriptome (23310 transcripts) in all eight samples with two biological replicates. Correlation analysis (coefficients R^2^) and hierarchical cluster analysis were performed using R software. Red color indicates a stronger correlation and green weaker (R^2^). **c** The number of differentially expressed genes (DEGs) resulting from pairwise comparisons of transcriptomes between Dd and Rd (Dd/Rd), Dd and Dh (Dd/Dh), Dh and Rh (Dh/Rh), Rd and Rh (Rd/Rh)

The transcriptomes of AZ-C from Dd, Rd, Dh, and Rh (Fig. 1a) were examined using the RNA-seq. Before RNA-sequencing analysis, *C*Las titer in AZ-C tissue was measured and the results verified that the fruits from HLB-diseased trees (Dd and Rd) were infected with *C*Las, while the fruits from healthy trees (Dh and Rh) were free of *C*Las (Fig. S1). Although the *C*Las titer in Dd was little bit higher than that in Rd, they were not statistically different (*p* > 0.05) (Fig. S1). Eight cDNA libraries were constructed and sequenced, two libraries for each of Dd, Rd, Dh, and Rh. The raw data have been deposited in NCBI Sequence Read Archive (SRA) through Gene Expression Omnibus (GEO) (access number: GSE101381). After filtering the low-quality and adapter sequences, a sequencing depth of 58–67 million 100 bp paired-end reliable reads per library was reached (Table S1). The clean reads were aligned to the reference genome sequences of *Citrus sinensis* v1.1^29^. The RNA-Seq reads were mapped to 21,781–22,452 *Citrus sinensis* transcripts. The mapped transcripts and the corresponding *Arabidopsis* orthologs as well as their annotations are listed in Table S2.

Hierarchical cluster analysis of the reads was conducted to evaluate the distances among samples based on correlations regarding gene expression, and the results indicate that gene expression correlations between the biological replicates had high coefficients (*R*^*2*^ = 0.982–0.989, *n* = 23,310) (Fig. 1b), demonstrating the reliability of the data produced and illustrating the consistency of the transcriptional changes within each type of fruit. Dd groups were farthest away from all other groups, indicating that the transcriptome profile in the AZ-C of dropped fruit from HLB-diseased trees (Dd) was quite different from all other types of fruit. Although both from HLB-diseased trees, the similarity between retained (Rd) and dropped fruit (Dd) was less than between HLB and healthy fruit (Rd vs. Rh or Dh). Meanwhile, the Dh groups were closer to Rh than to Dd or Rd, indicating that the dropped fruit from healthy trees (Dh) was more similar to retained fruit on healthy trees (Rh) than to HLB-affected fruit (Rd or Dd).

Pairwise comparisons between Dd and Rd, Dh and Rh, Dd and Dh, and Rd and Rh resulted in four sets of regulated genes, respectively, for Dd vs. Rd, Dh vs. Rh, Dd vs. Dh, and Rd vs. Rh. Those genes with a fold change of at least 2 (|log2FC|≥1), and with a *p*-value less than 0.05 were considered differentially expressed genes (DEGs). There were 1047 (403 up- and 644 downregulated), 1599 (777 up- and 822 downregulated), 813 (553 up- and 260 downregulated), and 764 (521 up- and 243 downregulated) DEGs, respectively, for Dd vs. Rd, Dd vs. Dh, Dh vs. Rh, and Rd vs. Rh (Fig. 1c).

### Significantly regulated functional gene groups

**Fig. 2:**
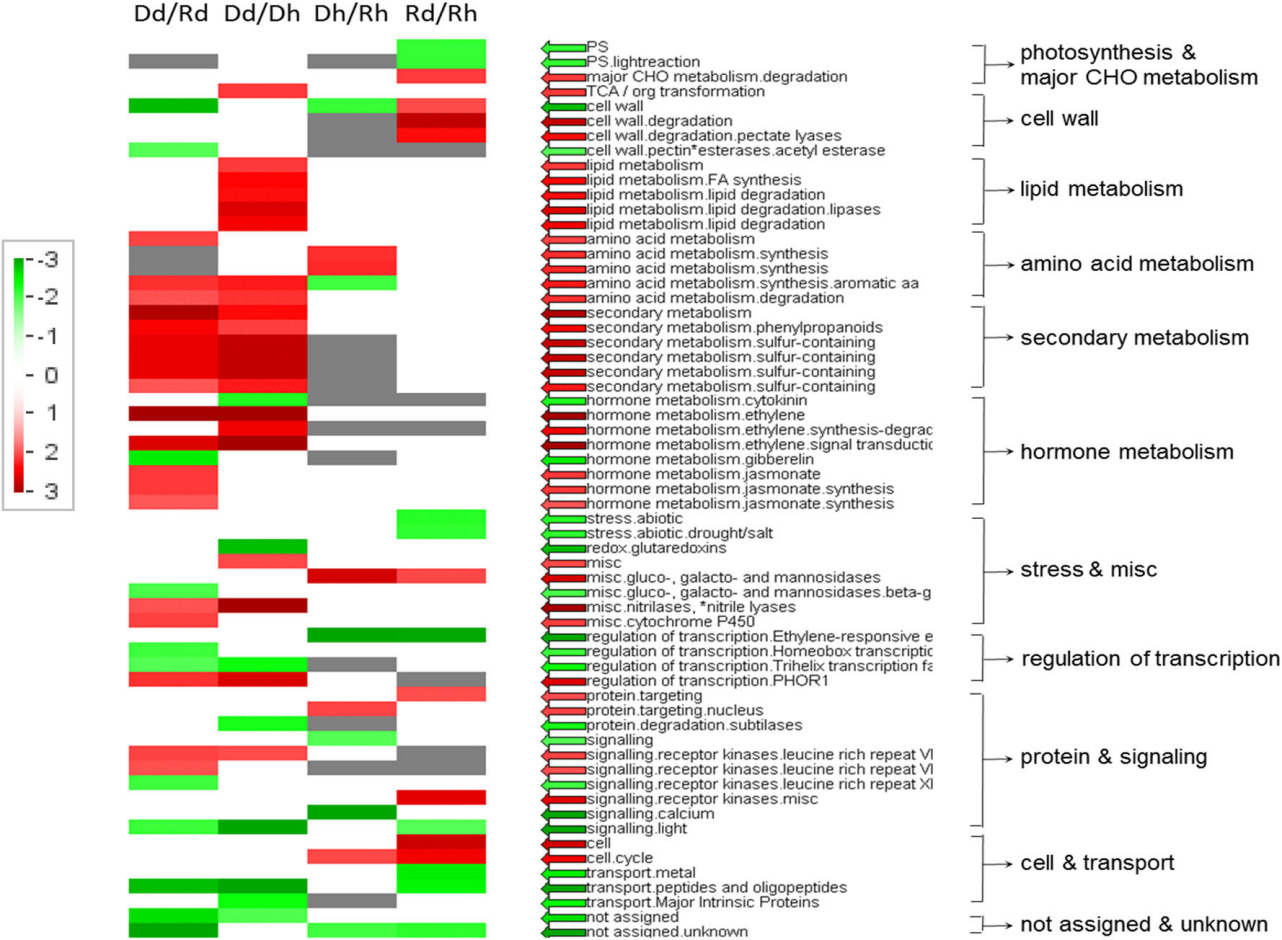
PageMan display of results of Bin-wise Wilcoxon rank sum test for significant MapMan gene categories that were regulated in calyx abscission zones of D compared to R (Dd vs Rd and Dh vs Rh) and counterparts between HLB-diseased and healthy (Dd vs Dh and Rd vs Rh). Colored boxes indicate statistically significant groups (*p*-value < 0.05). The color scale represents regulation of gene expression, with red indicating a trend within the group for upregulation of expression, and green, downregulation. The arrows shown with the same color scheme, and the text alongside each row, provide MapMan annotation of differentially regulated gene classes

To gain a holistic understanding of the functional groups that were involved in the abscission of HLB-affected fruit, the DEGs were subjected to Wilcoxon rank-sum test analysis by PageMan^30^ integrated in the MapMan software^31^. The functionally related groups of genes were identified, which showed significant patterns of regulation compared with the complete collection of genes under analysis. A graphical summary, illustrating differentially regulated functional groups, is shown in Fig. 2. DEGs in each group and the results of Wilcoxon test are listed in Table S3.

Although the total number of upregulated genes were less than that of downregulated genes in DEGs of Dd vs. Rd and Dd vs. Dh (Fig. 1c), more significantly upregulated than downregulated gene groups were identified by the Wilcoxon test (Fig. 2). The most noteworthy upregulated gene groups in AZ-C of Dd fruit include: lipid metabolism, amino acid and secondary metabolism, hormone metabolism–ET, and –jasmonate, signaling-receptor kinases, etc; while the most noteworthy downregulated gene groups in Dd were “transport” related and a group of non-annotated (unknown) genes (Fig. 2, lines Dd/Rd and Dd/Dh). Quite different from Dd, few gene groups regulated in Dh were identified by the test, with a mainly upregulated gene group related to amino acid metabolism (Fig. 2, line Dh/Rh). The comparison of Rd with Rh showed that gene groups related to major carbohydrate metabolism, cell wall degradation, and cell cycle were upregulated, while photosynthesis, abiotic stress, transport, and a group of functionally unknown genes were downregulated in Rd compared with Rh (Fig. 2, line Rd/Rh).

Since the goal of the study was to determine the mechanism of HLB-related fruit abscission, the analyses and discussions afterward will be focused on the comparison between dropped and non-dropped fruit from HLB-trees (Dd vs. Rd), and compared with those from healthy trees (Dh vs. Rh).

### Enrichment analyses of DEGs for the significant biological processes and pathways

**Fig. 3:**
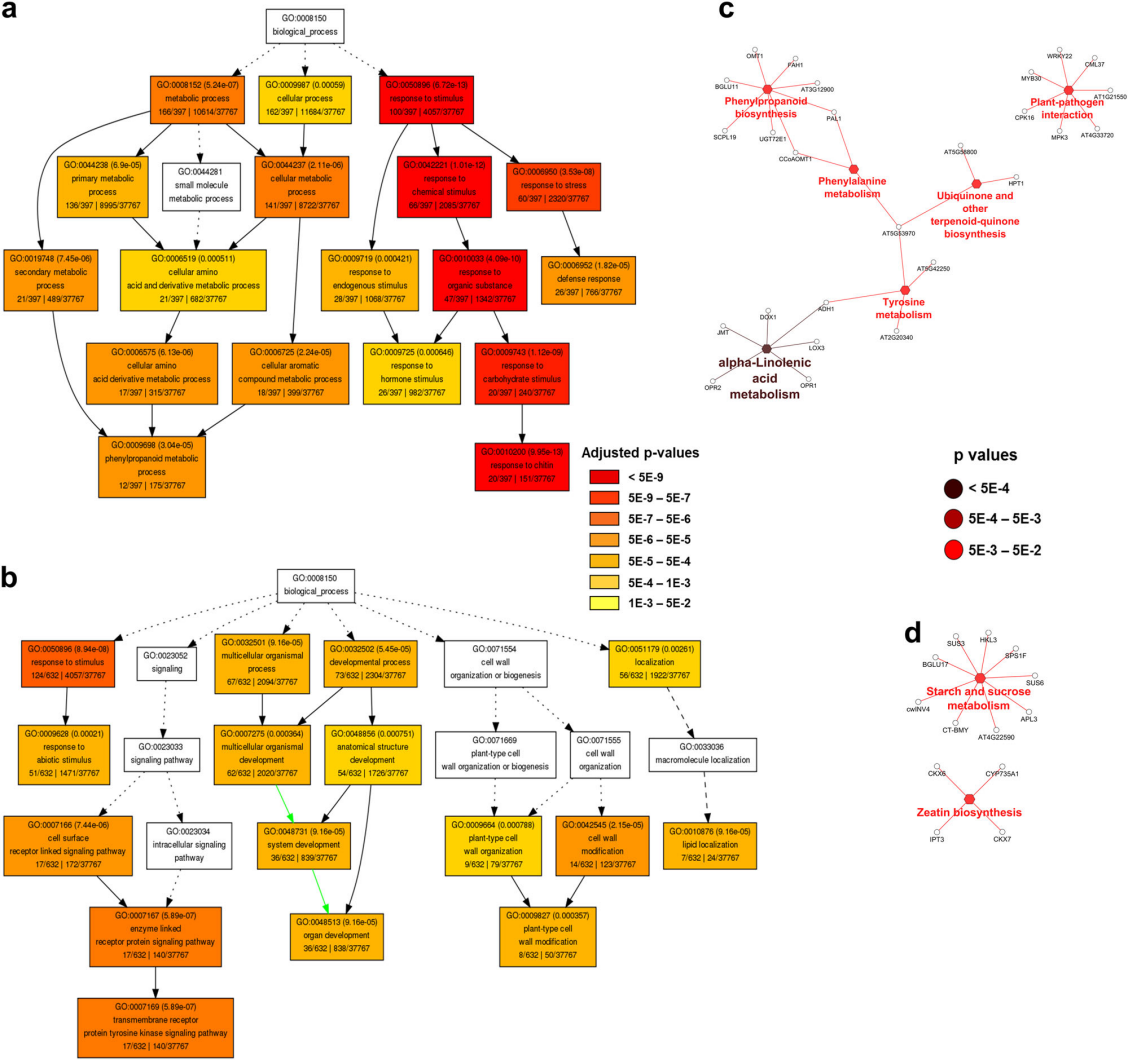
Diagrams showing significant terms by gene ontology (GO) and Kyoto Encyclopedia of Genes and Genomes (KEGG) pathway enrichment analyses of differentially expressed genes (DEGs) between dropped diseased (Dd) vs retained diseased (Rd). **a**, **b** Significant GO terms under the category of “Biological Process” for upregulated (**a**) and downregulated (**b**) genes. Statistical test method: hypergeometric, and multi-test adjustment method: Yekutieli (FDR under dependency). **c**, **d** The significant KEGG pathways related to upregulated (**c**) and downregulated genes (**d**). Statistical test method: two-sided hypergeometric test

**Fig. 4:**
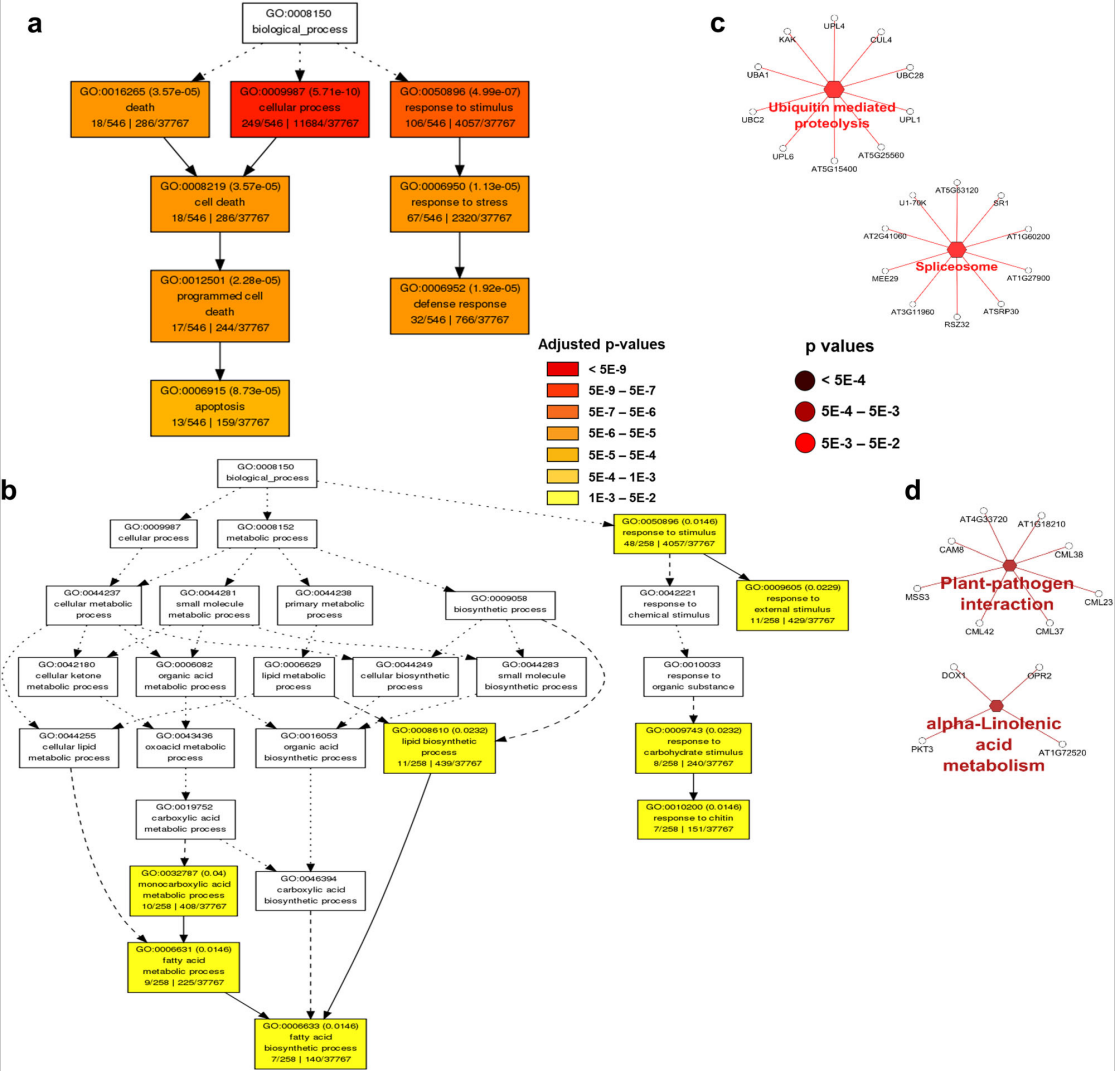
Diagrams showing significant terms by the gene ontology (GO) and Kyoto Encyclopedia of Genes and Genomes (KEGG) pathway enrichment analyses of differentially expressed genes (DEGs) between dropped healthy (Dh) vs. retained healthy (Rh). **a**, **b** Significant GO terms under the category of “Biological Process” for upregulated (**a**) and downregulated (**b**) genes. Statistical test method: hypergeometric, and multi-test adjustment method: Yekutieli (FDR under dependency). **c**, **d** The significant KEGG pathways related to upregulated (**c**) and downregulated genes (**d**). Statistical test method: two-sided hypergeometric test

To understand the significant biological processes and pathways that are involved in the HLB-related fruit drop, the upregulated and downregulated DEGs from the comparisons between dropped and retained fruit (Dd vs. Rd and Dh vs. Rh) were subjected to the gene ontology (GO) and KEGG pathway enrichment analyses, which are illustrated in Figs. 3 and 4, respectively for Dd vs. Rd and Dh vs. Rh. All the GO terms and KEGG pathways are listed in Table S4. For the GO enrichment results illustrated in Fig. 3a, b, and Fig. 4a, only the GO terms with multiple-test adjusted *p*-value below 0.001 (highlighted by colors) are shown due to the big numbers of GO terms with multiple-test adjusted *p*-value below 0.05.

For Dd (Fig. 3), the significantly upregulated biological process (Fig. 3a) were those related to defense response (GO:0006950, GO:0006952, GO:0050896, GO:0042221, GO:0010033, GO:0009743, and GO:0010200), secondary metabolism (GO:0019748, GO:0006575, GO:0006725, and GO:0009698), and hormone signaling (GO:0009719, GO:0009725). The significantly upregulated pathways (Fig. 3c) include plant hormone jasmonic acid biosynthesis (“alpha-linolenic acid metabolism” pathway (KEGG:00592, *p* = 4.6E-5)) and the pathways related to secondary metabolism and defense response to the pathogen (“phenylpropanoid biosynthesis” (KEGG:00940, *p* = 9.9E-3), “phenylalanine metabolism” (KEGG:00360, *p* = 4.3E-2), “ubiquinone and other terpenoid-quinone biosynthesis” (KEGG:00130, *p* = 2.7E-2), “tyrosine metabolism” (KEGG:00350, *p* = 6.3E-3), and “plant–pathogen interaction” (KEGG:04626, *p* = 3.5E-2)). Among these biological processes or pathways, “response to chitin” was the most significant biological process (multi-test adjusted *p* = 9.95E-13), and “alpha-linolenic acid metabolism” (jasmonic acid biosynthesis) was the most significant pathway (*p* = 4.6E-5) that were upregulated in Dd compared with Rd.

The significantly downregulated biological processes in Dd (Fig. 3b) were related to plant-type cell wall modification (GO:0042545, GO:0009664, and GO:0009827), cell surface receptor-linked signaling (GO:0007166, GO:0007167, and GO:0007169), organ development (GO:0032502, GO:0007275, GO:0048856, GO:0048731, and GO:0048513), and lipid localization (GO:0051179, GO:0010876); while the downregulated pathways in Dd (Fig. 3d) included “starch and sucrose metabolism” (KEGG:00500, *p* = 4.4E-2) and “zeatin biosynthesis” (KEGG:00908, *p* = 2.0E-2).

For Dh (Fig. 4), the significantly upregulated biological processes were related to cell death (GO:0016265, GO:0009987, GO:0008219, GO:0012501, and GO:0006915) and stress/defense response (GO:0050896, GO:0006950, and GO:0006952) (Fig. 4a); while the upregulated pathways (Fig. 4c) were related to protein degradation, including “ubiquitin-mediated proteolysis” (KEGG:04120, *p* = 6.4E-3) and transcription processing, including “spliceosome” (KEGG:03040, *p* = 4.7E-2). The downregulated biological processes for Dh (Fig. 4b) include “response to chitin” (GO:0010200) and GO terms related to lipid biosynthesis (GO:0008610, GO:0032787, GO:0006631, and GO:0006633). The downregulated pathways in Dh (Fig. 4d) include the “alpha-linolenic acid metabolism” pathway (KEGG:00592, *p* = 1.2E-3) and “plant–pathogen interaction” (KEGG:04626, *p* = 1.4E-3), both of which were contrarily upregulated in Dd (Fig. 3c).

Taken together, the enrichment analysis results indicated that defense response (especially “response to chitin”), secondary metabolism, and hormone signaling (in particular jasmonic acid) were upregulated (Fig. 3a, c), while organ development and cell wall organization were downregulated in Dd (Fig. 3b, d), which is quite different from its healthy tree counterpart Dh. In Dh (Fig. 4), “response to chitin” and jasmonic acid (JA) biosynthesis were downregulated, while “cell death” and protein degradation were upregulated. Cell death in Dh may be linked to wounding caused by the strong force applied to the healthy trees (in order to get enough numbers of “dropped fruit”). The cell wall degradation in Dd may be directly linked to the abscission process activated by plant hormones (such as JA) resulting from defense response.

The regulated genes under GO terms of “response to chitin” (GO:0010200) and “secondary metabolic process” (GO:0019748) are listed, respectively, in Table S5 and Table S6. Hormone-related genes will be further analyzed separately. Twenty chitin-responsive genes and 21 secondary metabolism-related genes were upregulated in Dd, but none of them were upregulated in Dh; and instead, some of them (six chitin-responsive genes and four secondary metabolism-related genes) were downregulated in Dh (Tables S5, S6). More than half (12 out of 21) of the secondary metabolism-related genes that were upregulated in Dd are from the phenylpropanoid pathway (Table S6, highlighted in red).

### Plant hormone-related gene expression

Plant hormones are known to mediate abscission signals^15^. The enrichment analyses showed “response to hormone stimulus” was the significant (*p* = 6.5E-4) biological process (Fig. 3a) and “jasmonic acid biosynthesis” (or “alpha-linolenic acid metabolism”) (*p* = 4.6E-5) was the significant pathway that were upregulated in Dd fruit (Fig. 3c). Therefore, the DEGs related to hormone biosynthesis and signaling were analyzed in detail, which are listed in Table S7. The profile of hormone gene expression was characterized as upregulation of ET and JA; and general downregulation of other hormones, including abscisic acid (ABA), auxin (AUX), brassinosteroid (BR), cytokinin (CK), and gibberellin (GA) in Dd, compared with Rd or Dh (Table S7). Different from ET and JA, salicylic acid (SA) was upregulated in dropped fruit from HLB-diseased trees (Dd) only when compared with dropped fruit from healthy trees (Dh), but not obviously regulated if compared with retained fruit on HLB-diseased trees (Rd) (Table S7).

The upregulation of ET in Dd included several 1-aminocyclopropane-1-carboxylate (ACC) oxidase genes (*ACO1*, *EFE* (or *ACO4*), and *MJM20.4* (or *ACO11*)) for ET biosynthesis and eight ERF genes (*ERF1*, *ERF4*, *ERF9*, *ERF13*, *ERF17*, *ERF20*, *ERF25*, and *ERF109*) for ET signaling. ACC synthase gene (*ACS*) expression was not regulated; however, gene expression of an ACS inhibitor, named “ethylene-overproduction protein” (ETO1), was downregulated. ETO1 is known to promote ACS degradation and inhibit ACS enzyme activity^32^. The upregulation of JA included genes for JA biosynthesis (*LOX3* (lipoxygenase 3 gene), *OPR1* and *OPR2* (12-oxophytodienoate reductase genes)), gene for methylation of jasmonate into methyljasmonate (*JMT*, jasmonate methyltransferase gene), and the JA-amino acid synthetase gene (*JAR1*). Moreover, gene expression of the key repressor of JA signaling, JAZ3 (jasmonate ZIM-domain protein 3)^33^, was downregulated in Dd fruit. The downregulation of ABA in Dd included the key genes for ABA biosynthesis (*NCED3* and *NCED4*) and two ABA-responsive protein genes (*HVA22E* and *GEM*) (Table S7).

**Fig. 5:**
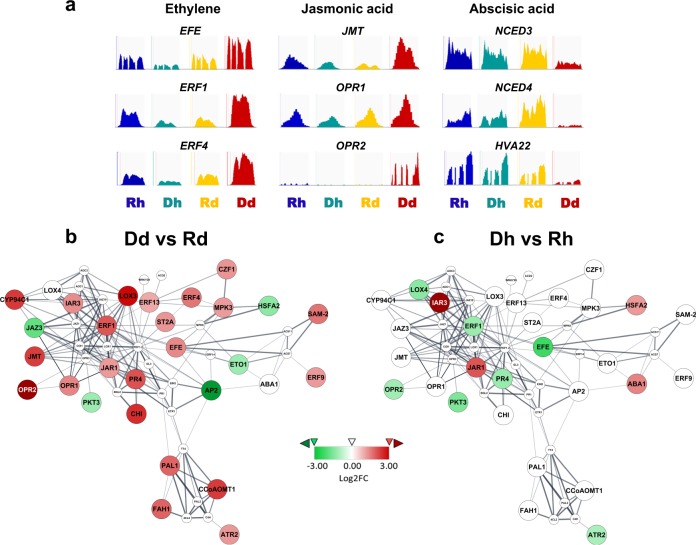
Gene expression level and protein-protein interaction network for gene products related to ethylene, jasmonic acid and abscisic acid pathways. **a** Gene expression level viewed by Integrative Genomics Viewer. **b**, **c** Protein–protein interaction network showing regulation of gene expression in the comparisons of Dd vs. Rd (**b**) or Dh vs. Rh (**c**). Red indicates the upregulated and green indicates the downregulated

Figure 5a shows gene expression levels (mapped reads) viewed by Integrative Genomics Viewer (IGV), for some example DEGs involved in pathways of ET, JA, or ABA.

Protein–protein interaction (PPI) network for the JA/ET pathway-related proteins was predicted based on the knowledge of PPI network of *Arabidopsis*^34^. The gene expression data (log2FC) for Dd vs. Rd and Dh vs. Rh were mapped to the PPI network, in which the regulated proteins/gene expression are illustrated by red or green color, indicating up- or downregulation, respectively; white indicates no change (Fig. 5b, c). The network includes the JA/ET pathway proteins that have been known to have interactions with each other, and proteins of other pathways/functional groups known to have connections with the JA/ET pathway proteins. For Dd vs. Rd (Fig. 5b), numerous genes of ET or JA synthesis or signaling pathways were upregulated, while genes for negative regulators of ET (*ETO1*) or JA (*JAZ3*) pathways were downregulated (Fig. 5b). Dh vs. Rh (Fig. 5c) was quite different from Dd vs. Rd, with only two genes (*IAR3* and *JAR1*) that were similarly regulated to Dd vs. Rd, which were upregulated in both Dd and Dh (Fig. 5b, c). IAR3 is an enzyme that hydrolyzes amino acid conjugates of IAA or JA, and is induced in response to wounding^35^. JAR1 is known to play a role in JA-mediated defense response^36^, and as well, is induced in response to wounding^37^. Also, shown in the network are several gene products from secondary metabolism pathways that have direct connections with ET/JA, among them, are PAL1, CCoAOMT1, FAH1, and ATR2, gene expressions of which were upregulated in Dd vs. Rd (Fig. 5b), but were not downregulated in Dh vs. Rh (Fig. 5c).

Consistent with the upregulation of ET and JA, substantial numbers of downstream JA/ET-responsive defense-related genes were upregulated in Dd compared with both Rd and Dh fruit (Table S8). These defense genes include plant defensin type 1 gene (*PDF1.4*)^38^, an antifungal chitin-binding protein gene called pathogenesis-related 4 (*PR4*, or *HEL*), polygalacturonase-inhibiting protein gene (*PGIP1*), beta-1,3-glucanase gene (*BG1*), sulfotransferase 2A gene (*ST2A*), alpha-dioxygenase 1 gene (*DOX1*), cysteine proteinase gene (*RD21A*), several chitinase genes (*CHIB1*, *CHIC*, and *CHIV*), and plant U-box protein genes (*PUB21*, *PUB22*, *PUB23*, *PUB24*, and *PUB29*). None of these defense response genes were upregulated in Dh compared with Rh. However, several (5 out of 21) of them were higher in HLB-affected fruit (Rd) compared with healthy fruit (Rh) (Table S8).

### Quantitative RT-PCR validation of DEGs

To verify the RNA-Seq data by qRT-PCR, 30 genes were selected as representatives covering each of the DEG categories identified by RNA-Seq analysis, including genes related to the seven categories of hormones (with an emphasis on ET and JA), secondary metabolism, and JA/ET-mediated defense responses (Fig. S2a). The qRT-PCR results for the 30 genes were comparable with the RNA-Seq results, with a squared correlation-coefficient value of 0.87 (Fig. S2b).

### Phytohormone production measurements

**Fig. 6:**
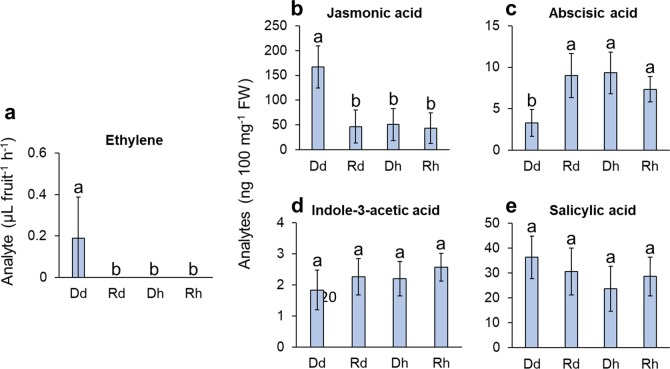
**Barplot showing phytohormone production by Dd, Rd, Dh, or Rh**

To verify the gene expression data at final product level, phytohormone ET, JA, abscisic acid (ABA), indole-3-acetic acid (IAA), and salicylic acid (SA) were measured. Gaseous ET was directly measured for individual fruit. Being a gaseous compound ET affects fruit and plant parts that are remote from site of synthesis, and fruit ET production has been shown to be a predictor of abscission^39,40^. Fruit ET production was detected in 39 out of 60 Dd fruit, but none of the Rd, Dh, or Rh fruit exhibited detectable ET production under the measurement conditions used (Fig. 6a). The average ET production of the 60 Dd fruit was 0.188 (µL fruit-1 h-1), and statistical analysis indicated that ET production from Dd fruit was significantly higher than the other three types of fruit (*p* = 3.84E-11), which had no ET detected (Fig. 6a).

Non-volatile acidic phytohormones JA, ABA, SA, and IAA were extracted from AZ-C tissue and derivatized to volatile methyl esters. The GC–MS results showed significantly higher level of JA (Fig. 6b) and lower level of ABA (Fig. 6c) in Dd compared with Rd, Dh, or Rh AZ-C tissue. IAA (Fig. 6d) and SA (Fig. 6e) showed no significant differences among Dd, Rd, Dh, and Rh; however, there was a trend where IAA was lower in Dd fruit and SA higher compared with Rd, Dh, or Rh.

### Detection of fungus Diplodia infection in AZ-C

Since the RNA-seq analysis revealed a gene expression profile of antifungal defense (upregulation of “response to chitin”, “secondary metabolism” and “JA/ET signaling”, and “JA/ET-activated defense response”) in AZ-C of Dd fruit, and a higher incidence of fungus Diplodia had been reported in the AZ-C of HLB-affected citrus fruit compared with healthy controls^27^, we tested Diplodia infection in AZ-C of Dd, Rd, Dh, and Rh fruit. The qPCR results (Fig. S3) showed that Diplodia level in Dd was significantly higher than in Rd, Dh, or Rh (*p* < 0.001); while there was no difference among Rd, Dh, and Rh (*p* > 0.05) (Fig. S3).

## Discussion

In this study, profiling of global gene expression in AZ-C revealed the upregulation of genes involved in ET and JA pathways, and JA/ET-activated defense response (of which the “response to chitin” was the most significant), as well as secondary metabolism in the HLB-affected fruit that were undergoing abscission (Dd) compared with those not undergoing abscission (Rd). But the counterparts from healthy trees showed different patterns of regulation for gene expression, which mainly related to “response to wounding”, “cell death”, and “protein degradation”. At the time of sampling, in order to get enough numbers of “dropped fruit” from healthy trees (Dh) for the experiments, we had to increase the force for shaking the healthy trees. One observation was that none of Dd had an attached calyx, because the calyx detached from Dd when Dd fruit abscised upon shaking the trees; however, the majority of Dh still had the calyx attached, similar to retained fruit (Rh and Rd) (Fig. 1a). So, most of the Dh fruit may not really be undergoing abscission, or at least not in the same way as the Dd fruit.

Abscission is often related to stresses (biotic or abiotic) and senescence, and the abscission signals are mediated by phytohormones. In a general sense, hormones such as ET and JA act as abscission-accelerating signals, and ABA promotes abscission through the actions of ET; while others such as AUX, GA, and BR act as abscission-inhibiting signals^15^. ET is the pivotal effector of abscission, and it responds to both biotic and abiotic stresses;^41^ while JA and ABA mediate abscission signals triggered by biotic and abiotic stress, respectively. JA is well known to be the central player of plant defense against necrotrophic fungi and herbivorous insects^42^; and contrary to JA, ABA is widely recognized as the primary hormonal signal of plant response to abiotic stressful conditions, such as dehydration, cold temperatures, or nutrient shortage^20,43^. In our study, both RNA-Seq data (Table S7) and GC–MS data (Fig. 6a–c) showed upregulation of ET and JA, while downregulation of ABA in Dd compared with Rd or Dh fruit, indicating that the abscission of Dd fruit was mediated by ET and JA signaling, and linked to biotic stress. The profile of hormone gene expression in Dd is similar to that of citrus postbloom fruit drop (PFD), which is caused by the infection of citrus petals with the fungus *C. acutatum*, and has been found to be associated with increased ET and JA in petals^19^.

JA has been reported to promote fruit abscission^44^ by inducing ET-forming enzyme gene (*EFE* or *ACO4*) expression^45^ or in an ET-independent manner by modifying saccharide metabolism in the abscission zone^46^; and ABA has been found to be involved in citrus organ abscission through ACC (1-aminocyclopropane-1-carboxylic acid)^20^. The citrus fruitlet abscission (June drop) caused by carbohydrate shortage^20^, and the abscission of citrus fruit or leaves induced by water stress^47^ were both associated with an increase in ABA, which consequently elevated the levels of ACC and ET, resulting in the plant organ abscission^2^. In our study, no ACC synthase gene (*ACS*) was found to be regulated in AZ-C of Dd fruit; but instead, multiple ACC oxidase genes (*ACO1*, *EFE* (or *ACO4*), and *MJM20.4* (or *ACO11*)) and ERF genes (*ERF*s) were significantly upregulated, which are known to be induced by JA^45,48^. This detailed information about regulation of ET in Dd agrees with the data for JA and ABA, which showed upregulation of JA and downregulation of ABA.

The biological processes "response to chitin" (GO:0010200) and “Phenylpropanoid metabolic process” (GO:0009698) were significantly (multi-test adjusted *p* = 9.95E-13 and 3.04E-5, respectively) upregulated in Dd fruit (Fig. 3a), both of which have been related to JA pathways^49,50^. Chitin, a major component of fungal cell walls, has been recognized as an elicitor of plant defense responses mediated by JA^49^; and the phenylpropanoids as well, have been reported to be positively regulated by JA and its derivative methyl jasmonate (MeJA), to induce the accumulation of PAL (phenylalanine ammonia-lyase, the enzyme that catalyzes the first step of the phenylpropanoid pathway)^50^. In this study, 20 chitin-responsive genes were induced, and 21 secondary metabolism-related genes were induced in Dd, including a subset of genes (12 genes) important for the synthesis of phenylpropanoids (Table S6, red-highlighted items).

JA/ET, a combination of JA and ET signaling (or the synergistical action of JA and ET), has been well known to be activated in response to necrotrophic pathogens^51,52^; and the key element in the integration of ET and JA signals is ET-response factor 1 (ERF1), which is known to be induced synergistically by ET and JA^48^. In our study, there are eight ERF genes that were upregulated in Dd (Table S7), including *ERF1*. Consistently, substantial numbers of the JA/ET-activated defense genes^53,54^ (Table S8) were upregulated in AZ-C of Dd fruit, including genes encoding antifungal plant defensin type 1 (*PDF1.4*)^38^, polygalacturonase-inhibiting protein (*PGIP1*), beta-1,3-glucanase (*BG1*), pathogenesis-related 4 (*PR4*, or *HEL*), alpha-dioxygenase 1 (*DOX1*), several chitinases (*CHIB1*, *CHIC*, and *CHIV*), and plant U-box proteins (*PUB21*, *PUB22*, *PUB23*, *PUB24*, and *PUB29*). The products of these genes are recognized for their antifungal properties^54^, among them, PDF1 has been widely used as a marker for the induction of the JA/ET-dependent defense-signaling pathway in response to fungal infection^55^. Meanwhile, both chitinases and beta-1,3-glucanase are known to be induced by fungal infection and play active antifungal roles by hydrolyzing chitin polymers or beta-1,3-glucan structures^56,57^, the two major structural components of fungal cell walls^58^.

The features of gene expression in AZ-C of the dropped fruit from HLB-diseased trees (Dd) were all consistently related to an antifungal defense response, suggesting a mechanism for the involvement of fungal infection in HLB-associated fruit drop as has been suggested in previous studies^14,15^. There are reports that HLB causes citrus trees to be more susceptible to other pathogens^27,59^, and the secondary infection by other pathogens could contribute to the symptoms of HLB. One such secondary infection that had been reported is the infection of citrus fruit by fungus Diplodia (an opportunistic fungal pathogen in the citrus grove), which had been found to be present in the AZ-C of HLB-affected fruit with a higher incidence compared with healthy controls^27^. In our study, Diplodia infection was therefore tested in Dd, Rd, Dh, and Rh fruit. In line with the gene expression profile, significantly higher (*p* < 0.001) levels of Diplodia were detected in the AZ-C of Dd than in Rd, Dh, or Rh fruit (Fig. S3). *C*Las titers in these fruit were also tested, and the results indicated that *C*Las levels in Dd were not statistically different from that in Rd, although Dd had a lower average Ct value than Rd (Fig. S1). The results indicate a more direct link between HLB-associated fruit drop and fungal infection than with *C*Las infection itself.

SA, a plant hormone that is known to activate defense response against biotrophic pathogens, was not found to be regulated in dropped fruit (Dd) compared with retained fruit on HLB-diseased trees (Rd); however, when compared with dropped fruit from healthy trees (Dh), several genes in SA biosynthesis pathway were upregulated in Dd (Table S7). The results indicate that SA may be involved in HLB disease, although not directly related to HLB-associated fruit abscission. The comparison of Rd with Rh showed an upregulation of cell wall degradation in Rd (Fig. 2, line Rd/Rh), which could contribute to fruit abscission, as well as the increased susceptibility of HLB-affected fruit to other pathogens.

**Fig. 7:**
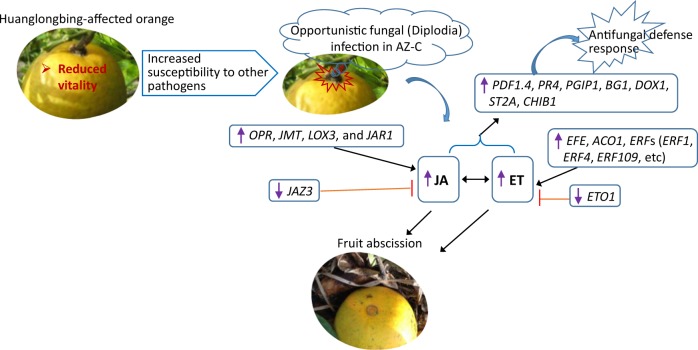
**Diagram illustrating regulation network involved in the citrus calyx abscission zone of huanglongbing-affected sweet orange**

Taken together, as summarized in Fig. 7, transcriptomic analysis revealed a gene expression profile in the abscission zone of Dd fruit that possesses typical characteristics of defense responses against necrotrophic fungal infection, manifested by upregulation of ET and JA, as well as activation of JA/ET-dependent antifungal defense response, and validated by phytohormone measurement and fungal detection. A previous study had showed higher incidence of infection by Diplodia in the AZ-C of HLB-affected fruit compared with healthy controls^27^, suggesting a secondary fungal infection at the fruit abscission zone. This study reveals the involvement of fungal infection in HLB-associated fruit drop, which includes inducing ET and JA production, and resulting in the activation of fruit abscission. The information will facilitate formulation of an effective strategy to control HLB-related fruit drop, perhaps by use of fungal controls in the field.

## Materials and methods

### Trees and fruit/tissue used in the experiments

Six-year-old “Hamlin” orange trees (Citrus sinensis (L.) Osbeck), about 2.5–3.0 -m tall, on “Swingle” citrumelo (C. paradisi Macf. × Poncirus trifoliata (L) Raf.) rootstock, were located in a commercial grove in Southern Florida. Eighteen *C*Las-positive (HLB-diseased) trees and 18 *C*Las-negative (healthy) trees were selected for the experiment. The presence or absence of *C*Las was tested by qPCR using the method of Li, et al.^60^. The selected trees were similar in size, and all were grown under similar agro-climatic conditions and received common cultural practices and the grower’s standard pest and disease management. Fruits were harvested on 1 December 2014 (during commercial harvest season). The ground under the trees was cleaned just before shaking the trees, and trees were shaken manually. For HLB-diseased trees (d), many of the fruit dropped (Dd) upon gentle shaking the trees. For healthy trees (h), the shaking was more aggressive in order to get enough dropped healthy fruits (Dh). The dropped fruits (Dd or Dh) were collected, and the retained fruits on HLB-diseased (Rd) or healthy trees (Rh) were harvested (Fig. 1a). The fruits from three trees were pooled together as a group, so there were six groups (field replicates) for each fruit category. From each group, ten fruits were randomly picked for RNA isolation and phytohormone extraction, another ten fruits were picked for ET measurement and DNA isolation. For the fruit used for RNA isolation and phytohormone extraction (60 fruits for each category of the Dd, Rd, Dh, and Rh), the AZ-C were excised (Fig. 1a) and immediately frozen in liquid nitrogen, then transported to the lab and stored at –80 °C. While the fruits used for ET measurement and DNA isolation (60 fruits for each of the Dd, Rd, Dh, and Rh) were transported to the laboratory where individual fruit ET production was measured, then AZ-C were excised for DNA isolation.

### Measurement of fruit ET production

Fruit ET production was determined by incubating individual fruit in 1-L glass jars, which were sealed for 1 h. One milliliter of headspace gas was withdrawn from each jar using a gas tight syringe and analyzed for ET by gas chromatography (Hewlett-Packard 5890, Avondale, PA) equipped with a flame ionization detector and an activated alumina column.

### DNA extraction and quantitative PCR (qPCR) detections

After the ET production measurement, AZ-C were excised and DNA was extracted for individual fruit using a DNeasy plant minikit (Qiagen, Inc., Valencia, CA) according to the manufacturer's instructions. DNA quality and quantity were assessed by spectrophotometry (NanoDrop; Thermo Scientific, Waltham, MA).

Real-time qPCR targeting *C*Las 16S rDNA was used for the titration of the *C*Las bacteria using the method described by Li et al.^60^; and qPCR targeting Diplodia β-tubulin gene was used for titration of fungus Diplodia infection in AZ-C tissue using the method described by Zhao et al.^27^.

### RNA extraction, cDNA library construction, sequencing, and data analysis

Eight RNA samples were prepared for cDNA library construction and sequencing. RNA was extracted from frozen AZ-C by RNeasy Plant Kit (Qiagen) following the manufacturer’s instructions. DNA was removed by on-column DNase treatment with RNase-free DNase Set (Qiagen). The purity and concentration of the total RNA were determined using a 2100 bioanalyzer (Agilent).

cDNA libraries were generated using the mRNA Sequencing Sample Preparation Kit following the manufacturer’s instructions (Illumina, CA, USA). Briefly, mRNA was purified from the total RNA using poly-T oligo-attached magnetic beads, and cDNA was then synthesized. cDNA fragments 200 ± 25 bp in size were selected, and PCR was performed with high-fidelity DNA polymerase, universal PCR primers and index (X) primer. PCR products were purified and the library preparations were sequenced on the Illumina cluster station and Illumina HiSeq1500 sequencing platform. The raw reads (fastq files) from Illunima HiSeq1500 were aligned to reference genome using TopHat version 1.4.1 with RefSeq annotations and the “—no-novel-juncs” option. Ambiguous reads that mapped to more than one region in the genome and those reads with a MAPQ score less than ten were removed. *Citrus sinensis* v1.1 from Phytozome v10.0 (https://phytozome.jgi.doe.gov/pz/portal.html#!info?alias=Org_Csinensis) was used as the reference genome and for transcript annotations. Transcript quantification was performed using Partek Genomics Suite (Partek Inc, St. Louis, MO), and the normalized read counts (RPKM: reads per kilobase per million mapped reads) were used to estimate the gene expression levels.

Differential expression analysis between treatments was performed using the DESeq R package. The resulting *p*-values were adjusted using the Benjamini and Hochberg’s approach for controlling the false-discovery rate (FDR). The significance of the gene expression difference was determined with a *p*-value < 0.05 found by DESeq. Transcripts with fold change over 2 and a *p*-value less than 0.05 were considered significant differential expression. BLAST search was used to identify the best *Arabidopsis* hit corresponding to each *C. sinensis* transcript.

### Hierarchical cluster analysis

Distance was based on Pearson correlation. The hierarchical clustering and other statistical analyses were performed using R/Bioconductor (http://www.r-project.org).

### Functional categorization

The differentially expressed genes (DEGs) were functionally analyzed with the MapMan software^31^ by mapping DEGs to the TAIR database. Log2 fold change values (log2FC) of the whole set of DEGs were subjected to the Wilcoxon rank-sum test analysis, and the results were visualized using PageMan^30^ application that is integrated in the MapMan.

### Enrichment analyses

The gene ontology (GO) enrichment analyses of the up- or downregulated DEGs were performed using AgriGO (http://bioinfo.cau.edu.cn/agriGO/), a bioinformatics platform especially for agricultural community^61^. Significant GO terms were identified using a hypergeometric test with a Yekutieli (FDR under dependency) correction. Because of big numbers of GO terms turning out, only the GO terms with top significance (multiple-test adjusted *p*-value < 0.001) are shown in the graph and used for further analyses. KEGG enrichment analysis was conducted by using ClueGO^62^ plug-in of Cytoscape software^63^. Significant pathways were identified using a two-sided hypergeometric test with a significance threshold of 0.05.

### PPI network

A predicted PPI network was constructed for Citrus based on PPIs in *Arabidopsis*^34^ for the DEGs of Dd vs. Rd and Dh vs. Rh. Networks were identified and visualized using Cytoscape software^63^. Nodes of the network represented proteins encoded by DEGs and their functional partners in the predicted pairwise interaction network.

### Quantitative RT-PCR (qRT-PCR) validation

The total RNA was extracted from frozen AZ-C by RNeasy Plant Kit (Qiagen) following the manufacturer’s instructions. DNA was removed by on-column DNase treatment with RNase-free DNase Set (Qiagen). The RNA quality and quantity were detected with a Nanodrop spectrophoto-meter (Thermo Scientific, USA). cDNA synthesis was performed using SuperScript^®^ VILO™ cDNA Synthesis Kit (Life Technologies). Gene-specific primers were designed using software Primer Express 3.0.1 (Applied Biosystems). The primer sequences are listed in Table S9. Real-time PCR amplifications were performed in a 7500 real-time PCR system (Applied Biosystems, Foster City, CA). The qPCR parameters were as follows: 95 °C for 10 min, followed by 40 cycles at 95 °C for 15 s, and 60 °C for 1 min, with fluorescence signal capture at each stage of 60 °C. The default Melt Curve (disassociation) stage was continued after the 40 cycles of PCR. Cycle-threshold (Ct) values are analyzed using ABI 7500 Software version 2.0.6 (Applied Biosystems, Inc., Carlsbad, CA) with a manually set threshold at 0.05 and automated baseline settings. Relative fold differences were calculated based on the comparative Ct (threshold constant) method using actin as an endogenous control. To determine relative fold differences for each sample, the Ct value for each gene was normalized to the Ct value for citrus actin^64^ and was calculated relative to a calibrator using the comparative Ct method (2^−ΔΔCt^).

### Measurement of non-volatile acidic phytohormone by GC–MS

Triplicated samples for each of Dd, Rd, Dh, and Rh were processed for extraction and analysis of JA, ABA, IAA, and SA. Frozen AZ-C tissue, 80 mg per sample, was ground to fine powder with a mortar and pestle, then used for vapor-phase extraction and GC–MS analysis as descripted by Schmelz et al.^65^. Briefly, powdered frozen tissue was homogenized and extracted in 300 µl of H_2_O:1-propanol:HCl (1:2:0.005) and l ml of dichloromethane, by using a bead-beater (Omni International, Kennesaw, GA). After centrifugation, the organic phase was transferred to a glass vial and derivatized to methyl esters (MEs) with 2 µl of trimethylsilyldiazomethane (Sigma-Aldrich). Methylated compounds in this mixture were volatilized by heating in the presence of a N_2_ stream, and collected on a volatile collection trap (Analytical Research Systems, Gainesville, FL). Compounds were eluted from the volatile collection trap resin with dichloromethane and subsequently analyzed by GC–MS.

The GC–MS system consisted of a 6890 Network GC connected to a 5973 inert Mass Selective Detector (Agilent, Palo Alto, CA). Compounds were separated on a DB5MS column (60 m × 0.25 mm × 0.25 μm). The temperature regime for GC was 40 °C for 1 min after injection, followed by sequential temperature ramps of 15 °C/min to 250 °C, and then maintained the temperature for 10 min. The identities of JA, ABA, IAA, and SA in the tissue samples were confirmed by comparison of the elution time and mass spectra to authentic chemical standard compounds (Sigma-Aldrich). The acid ME/TMS analyses were measured using SIM with retention times and m/z ion as follows: JA (M – trans 16.12/cis 16.46 min, 224), ABA (M – 21.61 min, 278), SA (M – 12.64 min, 152), IAA (M – 17.23 min, 189).

## Supplementary information

Fig. S1. Boxplot showing CLas Ct values tested by qPCR for Dd, Rd, Dh, and Rh. n=60

Fig. S2. **a**. Results of qRT-PCR analysis of 30 DEGs identified by RNA-Seq analysis in the comparison Dd with Rd. Data represents Mean ± SEM of 3 independent experiments. **b**. Correlation between results of qRT-PCR and RNA-Seq analysis

Fig. S3. Boxplot showing Diplodia Ct values tested by qPCR for Dd, Rd, Dh, and Rh. n=60

Table S1. RNA-Seq reads and mapping summary

Table S2. List of mapped transcripts

Table S3 a-h MapMan annotation of differentially expressed genes (DEGs) and results of Wilcoxon Rank Sum Test of gene classes

Table S4a-h List of Biological_Process Gene Ontology terms and KEGG pathways in enrichment analysis and their *p* values

Table S5. Genes induced in response to chitin

Table S6. Genes involved in secondary metabolism (genes from phenylpropanoid pathway are highlighted in red)

Table S7. List of regulated phytohormone genes

Table S8. JA/ET induced defense response genes

Table S9. Genes and the primers used in the qRT-PCR

## Data Availability

The datasets supporting the conclusions of this article are included within the article and its additional files. The raw data of RNA Sequencing have been deposited in NCBI Sequence Read Archive (SRA) through Gene Expression Omnibus (GEO) (access number: GSE101381).
