# Supplementary figures and images for: Comparative analysis of the transcriptomes of the calyx abscission zone of sweet orange insights into the huanglongbing-associated fruit abscission
Source: Hortic Res. 2019 Jun 1;6:71. doi: 10.1038/s41438-019-0152-4 (PMC6544638; doi:10.1038/s41438-019-0152-4)

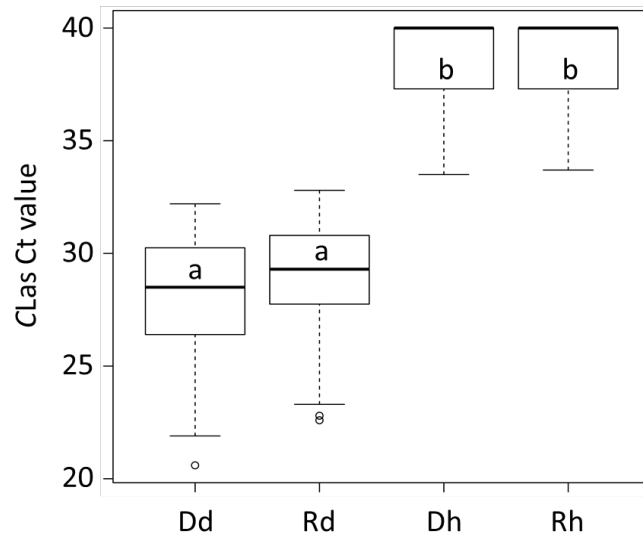

Fig. S1. Boxplot showing Clas Ct values tested by qPCR for Dd, Rd, Dh, and Rh. n=60.

Supplement: Supplementary file 1 — Fig. S1. Boxplot showing CLas Ct values tested by qPCR for Dd, Rd, Dh, and Rh. n=60 [file 41438_2019_152_MOESM1_ESM.pdf]

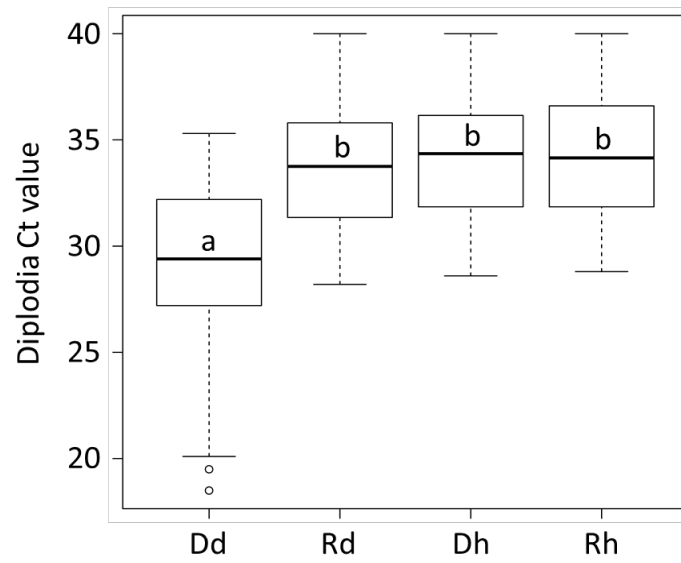

Fig. S3. Boxplot showing Diplodia Ct values tested by qPCR for Dd, Rd, Dh, and Rh. n=60.

Supplement: Supplementary file 3 — Fig. S3. Boxplot showing Diplodia Ct values tested by qPCR for Dd, Rd, Dh, and Rh. n=60 [file 41438_2019_152_MOESM3_ESM.pdf]
