# Supplementary material for: Comparative analysis of the transcriptomes of the calyx abscission zone of sweet orange insights into the huanglongbing-associated fruit abscission
Source: Hortic Res. 2019 Jun 1;6:71. doi: 10.1038/s41438-019-0152-4 (PMC6544638; doi:10.1038/s41438-019-0152-4)
Supplement: Supplementary file 2 — Fig. S2. a. Results of qRT-PCR analysis of 30 DEGs identified by RNA-Seq analysis in the comparison Dd with Rd. Data represents Mean ± SEM of 3 independent experiments. b. Correlation between results of qRT-PCR and RNA-Seq analysis [file 41438_2019_152_MOESM2_ESM.pdf]

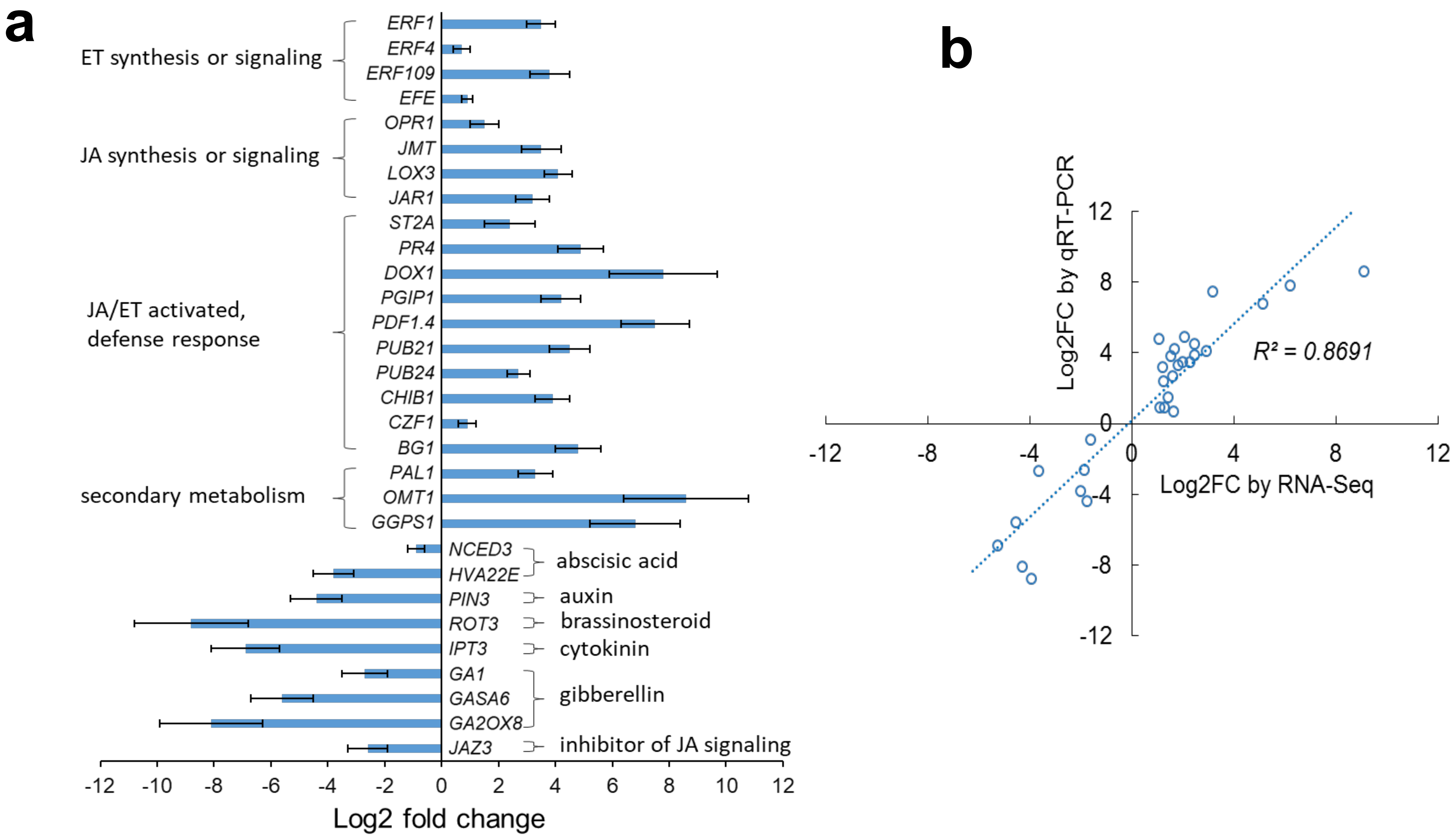

Fig. S2. a. Results of qRT-PCR analysis of 30 DEGs identified by RNA-Seq analysis in the comparison Dd with Rd. Data represents Mean ± SEM of 3 independent experiments. b. Correlation between results of relative gene expression from qRT-PCR and from RNA-Seq analysis.
