# Supplementary material for: Comparative analysis of the transcriptomes of the calyx abscission zone of sweet orange insights into the huanglongbing-associated fruit abscission
Source: Hortic Res. 2019 Jun 1;6:71. doi: 10.1038/s41438-019-0152-4 (PMC6544638; doi:10.1038/s41438-019-0152-4)
Supplement: Supplementary file 4 — Table S1. RNA-Seq reads and mapping summary [file 41438_2019_152_MOESM4_ESM.pdf]

Table S1. RNA-Seq reads and mapping summary

Library	Number of Reads	Number of reliable Reads	Number of Alignments	Number of mapped transcripts
Rh1	75,286,011	65,721,812	124,048,355	22,294
Rh2	72,376,606	60,856,282	94,444,596	21,890
Dh1	72,456,154	60,551,924	94,738,453	22,001
Dh2	76,570,404	64,541,823	101,110,349	22,100
Rd1	77,807,586	67,126,125	127,058,842	22,452
Rd2	75,673,616	63,231,082	98,969,996	22,107
Dd1	78,922,586	67,538,888	127,074,654	22,149
Dd2	71,799,017	58,926,095	91,854,338	21,781
