# Supplementary material for: Comparative analysis of the transcriptomes of the calyx abscission zone of sweet orange insights into the huanglongbing-associated fruit abscission
Source: Hortic Res. 2019 Jun 1;6:71. doi: 10.1038/s41438-019-0152-4 (PMC6544638; doi:10.1038/s41438-019-0152-4)
Supplement: Supplementary file 8 — Table S5. Genes induced in response to chitin [file 41438_2019_152_MOESM8_ESM.pdf]

Table S5. Genes induced in response to chitin

Citrus Gene ID	Best arabidopsis hit name	Dd/Rd		Dh/Rh		Gene symbol or description
		Log2FC	P value	Log2FC	P value	
orange1.1g012196m.g	AT4G33050	3.7	2.94E-02	-	-	*EDA39* , calmodulin-binding family protein
orange1.1g015513m.g	AT2G35930	2.26	4.09E-40	-1.07	8.76E-05	*PUB23* , plant U-box 23
orange1.1g040287m.g	AT2G37430	2.65	1.79E-14	-1.2	4.74E-02	*ZAT11,* zinc finger transcription factor
orange1.1g006236m.g	AT2G40140	1.26	1.21E-12	-	-	*CZF1* , zinc finger (CCCH-type) protein
orange1.1g032263m.g	AT2G44840	1.01	4.94E-02	-	-	*ERF13* , ethylene response factor 13
orange1.1g041408m.g	AT3G11840	1.59	6.35E-13	-	-	*PUB24* , plant U-box 24
orange1.1g028454m.g	AT3G15210	1.62	8.76E-07	-	-	*ERF4* , ethylene response factor 4
orange1.1g033041m.g	AT3G16720	1.67	8.09E-08	-	-	*ATL2* , ring-H2 finger protein
orange1.1g015939m.g	AT3G18710	1.23	2.18E-17	-	-	*PUB29* , plant U-box 29
orange1.1g024484m.g	AT3G23250	1.05	4.18E-07	-	-	*MYB15* , transcription factor
orange1.1g025683m.g	AT3G45640	1.18	1.68E-02	-	-	*MPK3* , mitogen-activated protein kinase 3
orange1.1g014913m.g	AT3G52450	1.93	1.17E-46	-1.13	3.16E-05	*PUB22* , plant U-box 22
orange1.1g021896m.g	AT4G01250	1.15	1.85E-08	-	-	*WRKY22* , transcription factor
orange1.1g028566m.g	AT4G17500	1.99	2.93E-12	-1.17	1.10E-03	*ERF1* , ethylene response factor 1
orange1.1g045327m.g	AT1G71520	1.13	3.83E-02	-	-	*ERF20* , ethylene response factor 20
orange1.1g042755m.g	AT4G34410	1.52	5.85E-08	-2.05	1.79E-12	*ERF109* , ethylene response factor 109
orange1.1g040502m.g	AT4G37260	1.19	4.25E-06	-	-	*MYB73* , transcription factor
orange1.1g038250m.g	AT5G37490	2.45	5.74E-46	-1.03	2.56E-04	*PUB21* , plant U-box 21
orange1.1g008967m.g	AT5G56960	1.47	9.88E-04	-	-	*bHLH* , basic helix-loop-helix transcription factor
orange1.1g045745m.g	AT5G59550	1.16	3.06E-18	-	-	*RDUF2* , zinc finger protein
