# Supplementary material for: Comparative analysis of the transcriptomes of the calyx abscission zone of sweet orange insights into the huanglongbing-associated fruit abscission
Source: Hortic Res. 2019 Jun 1;6:71. doi: 10.1038/s41438-019-0152-4 (PMC6544638; doi:10.1038/s41438-019-0152-4)
Supplement: Supplementary file 9 — Table S6. Genes involved in secondary metabolism (genes from phenylpropanoid pathway are highlighted in red) [file 41438_2019_152_MOESM9_ESM.pdf]

Table S6. Genes involved in secondary metabolism (genes from phenylpropanoid pathway are highlighted in red)

Citrus Gene ID	Best arabidopsis hit name	Dd/Rd		Dh/Rh		Gene symbol or description
		Log2FC	p value	Log2FC	p value	
**orange1.1g016039m.g**	**AT2G37040**	**1.82**	**3.85E-29**	**-**	**-**	***PAL1* , PHE ammonia lyase 1**
orange1.1g009152m.g	AT5G05260	3.35	1.52E-06	-	-	*CYP79A2* , phenylalanine N-monooxygenase-like protein
**orange1.1g043449m.g**	**AT5G54160**	**9.11**	**7.24E-36**	**-**	**-**	***OMT1* , O-methyltransferase 1**
**orange1.1g025602m.g**	**AT1G22640**	**1.82**	**1.51E-02**	**-**	**-**	***MYB3* , myb domain protein 3**
orange1.1g042896m.g	AT1G24100	1.57	3.45E-03	-	-	*UGT74B1* , UDP-glucosyl transferase 74B1
**orange1.1g040624m.g**	**AT1G49390**	**3.01**	**4.93E-09**	**-**	**-**	**2-oxoglutarate (2OG) and Fe(II)-dependent oxygenase**
**orange1.1g024461m.g**	**AT1G64520**	**3.59**	**2.07E-02**	**-**	**-**	***RPN12a* , regulatory particle non-ATPase 12A**
orange1.1g023929m.g	AT2G29420	1.93	7.26E-44	-1.63	4.08E-26	*GSTU7* , glutathione S-transferase tau 7
orange1.1g027956m.g	AT2G30860	1.07	1.74E-17	-	-	*GSTF9* , glutathione S-transferase PHI 9
orange1.1g040120m.g	AT1G10370	1.15	2.09E-04	-	-	*ERD9* , Glutathione S-transferase family protein
**orange1.1g038932m.g**	**AT3G29590**	**1.13**	**1.54E-02**	**-**	**-**	***AT5MAT* , HXXXD-type acyl-transferase**
orange1.1g025683m.g	AT3G45640	1.18	1.68E-02	-	-	*MPK3* , mitogen-activated protein kinase 3
**orange1.1g041419m.g**	**AT3G50740**	**1.77**	**1.32E-08**	**-1.35**	**3.57E-04**	***UGT72E1* , UDP-glucosyl transferase 72E1**
**orange1.1g005072m.g**	**AT4G30210**	**1.22**	**1.34E-16**	**-1**	**3.54E-09**	***ATR2* , P450 reductase 2**
orange1.1g010727m.g	AT4G31500	2.95	2.25E-40	-	-	*CYP83B1* , cytochrome P450, family 83, subfamily B, polypeptide 1
**orange1.1g029536m.g**	**AT4G34050**	**2.32**	**2.89E-11**	**-**	**-**	***CCoAOMT1* , S-adenosyl-L-methionine-dependent methyltransferases**
**orange1.1g038070m.g**	**AT4G36220**	**1.83**	**6.63E-05**	**-**	**-**	***FAH1* , ferulic acid 5-hydroxylase 1**
**orange1.1g018221m.g**	**AT1G17020**	**1.3**	**3.98E-08**	**-**	**-**	***SRG1* , senescence-related gene 1**
orange1.1g003146m.g	AT1G20780	1.13	3.42E-08	-	-	*SAUL1* , senescence-associated E3 ubiquitin ligase 1
orange1.1g012276m.g	AT5G09640	1.03	2.55E-14	-	-	*SCPL19* , serine carboxypeptidase-like 19
**orange1.1g031369m.g**	**AT5G14700**	**1.24**	**9.00E-05**	**-1.31**	**4.39E-04**	**NAD(P)-binding Rossmann-fold protein**
