# Supplementary material for: Comparative analysis of the transcriptomes of the calyx abscission zone of sweet orange insights into the huanglongbing-associated fruit abscission
Source: Hortic Res. 2019 Jun 1;6:71. doi: 10.1038/s41438-019-0152-4 (PMC6544638; doi:10.1038/s41438-019-0152-4)
Supplement: Supplementary file 10 — Table S7. List of regulated phytohormone genes [file 41438_2019_152_MOESM10_ESM.pdf]

Table S7. List of regulated phytohormone genes

Phytohormone Category	Citrus Gene ID	Best arabidopsis hit name	Dd/Rd		Dd/Dh		Dh/Rh		Rd/Rh		Gene symbol or description
			Log2FC	P value	Log2FC	P value	Log2FC	P value	Log2FC	P value	
Ethylene	orange1.1g046101m.g	AT3G12900	9.607133	9.31912E-86	9.490327	6.78858E-81	-	-	-	-	2-oxoglutarate (2OG) and Fe(II)-dependent oxygenase, *MJM20.4* , involved in ethylene synthesis
	orange1.1g042664m.g	AT5G54000	1.097108	0.037748733	2.482317	0.00010916	-	-	-	-	2-oxoglutarate (2OG) and Fe(II)-dependent oxygenase, *ACO* ,
	orange1.1g020953m.g	AT1G05010	1.388514	9.66226E-31	2.169168	9.88626E-26	-2.26777	0.039415273	-	-	ethylene-forming enzyme, *EFE*
	orange1.1g028454m.g	AT3G15210	1.622192	8.76039E-07	2.444697	7.06966E-10	-	-	-	-	ethylene responsive element binding factor 4, *ERF4*
	orange1.1g026240m.g	AT5G44210	1.197999	3.59226E-16	2.030151	6.34797E-13	-	-	-	-	ethylene responsive element binding factor 9, *ERF9*
	orange1.1g028566m.g	AT4G17500	1.989406	2.93154E-12	2.298938	5.60996E-15	-1.17135	0.00110325	-	-	ethylene responsive element binding factor 1, *ERF1*
	orange1.1g042755m.g	AT4G34410	1.521641	5.85138E-08	2.016971	7.72449E-12	-2.04975	1.79058E-12	-1.96166	5.40908E-09	ethylene responsive element binding factor 109, *ERF109*
	orange1.1g032263m.g	AT2G44840	1.008252	0.049427376	-	-	-	-	-	-	ethylene-responsive element binding factor 13, *ERF13*
	orange1.1g029068m.g	AT1G19210	1.38391	2.28441E-17	2.034013	1.53917E-08	-1.19866	0.024333411	-	-	ethylene-responsive element binding factor 17, *ERF17*
	orange1.1g045327m.g	AT1G71520	1.127225	0.038325779	3.32223	2.66682E-06	-	-	-	-	ethylene-responsive element binding factor 20, *ERF20*
	orange1.1g028004m.g	AT5G52020	1.532979	1.01303E-28	1.674738	7.44541E-20	-1.72556	1.2743E-23	-1.61502	5.71788E-32	ethylene-responsive element binding factor 25, *ERF25*
	orange1.1g002379m.g	AT3G51770	-1.16735	0.002179904	-1.6892	0.003810988	-	-	-	-	ethylene-overproduction protein 1, *ETO1*
Jasmonic Acid	orange1.1g007688m.g	AT1G17420	2.92333	0.000117702	3.157688	2.50151E-05	-	-	-	-	lipoxygenase 3, *LOX3*
	orange1.1g017448m.g	AT1G76680	1.43146	4.45534E-15	1.069347	1.01042E-09	-	-	-1.03528	5.23017E-08	12-oxophytodienoate reductase 1, *OPR1*
	orange1.1g017733m.g	AT1G76690	5.818177	1.1149E-160	5.43807	5.7774E-144	-1.09789	0.022869346	-1.50768	0.001214646	12-oxophytodienoate reductase 2, *OPR2*
	orange1.1g038593m.g	AT1G19640	2.285144	0.024132712	-	-	-	-	-	-	jasmonic acid carboxyl methyltransferase, *JMT*
	orange1.1g007464m.g	AT2G46370	1.194206	0.000183886	1.212279	0.009528555	2.010733	2.83888E-07	1.694568	2.98821E-05	jasmonic acid-amido synthetase, *JAR1*
	orange1.1g017322m.g	AT3G17860	-1.84865	0.027512776	-	-	-	-	-	-	jasmonate-zim-domain protein 3, *JAZ3*
Salicylic Acid	orange1.1g014245m.g	AT3G09830	-	-	1.085146	8.99409E-11	-	-	-	-	Protein kinase superfamily protein, *PCRK1*
	orange1.1g048393m.g	AT1G05680	-	-	1.531388	9.39127E-13	-	-	-	-	Uridine diphosphate glycosyltransferase 74E2, *UGT74E2*
	orange1.1g043411m.g	AT5G38020	3.021743	2.53167E-08	2.73564	6.00064E-07	-1.72098	0.012442162	-2.03949	0.002453638	S-adenosyl-L-methionine-dependent methyltransferases superfamily protein
	orange1.1g012382m.g	AT2G40000	-	-	1.342869	5.15903E-06	-	-	-	-	ortholog of sugar beet HS1 PRO-1 2, *HSPRO2*
	orange1.1g023929m.g	AT2G29420	1.926339	7.25978E-44	2.852345	2.71597E-85	-1.63057	4.077E-26	-	-	glutathione S-transferase tau 7, *GSTU7*
Abscisic Acid	orange1.1g007379m.g	AT3G14440	-1.61612	3.86079E-15	-1.58947	9.51156E-07	-	-	-	-	nine-cis-epoxycarotenoid dioxygenase 3, *NCED3*
	orange1.1g044599m.g	AT4G19170	-3.60908	1.49903E-26	-2.85522	1.57171E-12	-	-	1.025156	6.16085E-05	nine-cis-epoxycarotenoid dioxygenase 4, *NCED4*
	orange1.1g032264m.g	AT5G50720	-2.02234	4.16269E-15	-2.54286	3.73026E-18	-	-	-	-	HVA22 homologue E, *HVA22E*
	orange1.1g027849m.g	AT2G22475	-1.72808	3.01972E-05	-2.48248	3.10941E-12	-	-	-	-	GRAM domain family protein, *GEM*
Auxin	orange1.1g006199m.g	AT1G70940	-1.76634	3.43732E-08	-1.70671	7.38093E-24	2.149927	1.60581E-14	2.430281	4.35769E-07	Auxin efflux carrier family protein, *PIN3*
	orange1.1g009299m.g	AT2G26170	-1.22418	3.55924E-09	-1.30116	1.10334E-08	-	-	-	-	cytochrome P450, family 711, subfamily A, polypeptide 1, *CYP711A1*
	orange1.1g001093m.g	AT2G34680	-1.05305	2.50818E-09	-1.37016	2.12998E-15	2.50889	2.92138E-05	1.076563	0.002775155	Outer arm dynein light chain 1 protein, *AIR9*
	orange1.1g021436m.g	AT3G25290	-2.33624	1.58538E-07	-2.60273	8.13512E-10	-	-	-	-	Auxin-responsive family protein
	orange1.1g034194m.g	AT5G53590	-1.89547	0.000836275	-1.73917	0.003016436	-	-	-	-	SAUR-like auxin-responsive protein family
	orange1.1g039975m.g	AT4G38840	-2.79083	2.60377E-06	-2.2556	9.52071E-05	-	-	-	-	SAUR-like auxin-responsive protein family
Brassinosteroid	orange1.1g046307m.g	AT5G38970	-1.32968	4.60423E-06	-	-	-	-	-	-	brassinosteroid-6-oxidase 1, *BR6OX1*
	orange1.1g024678m.g	AT5G16010	-1.22635	0.004535108	-1.74586	6.65236E-06	-	-	-	-	3-oxo-5-alpha-steroid 4-dehydrogenase
	orange1.1g036815m.g	AT2G01950	-2.80677	0.001024437	-3.18997	4.31159E-05	-	-	-	-	BRI1-like 2, *BRL2*
	orange1.1g011403m.g	AT4G36380	-3.92335	3.74032E-78	-4.25012	5.40375E-97	-	-	-	-	Cytochrome P450 superfamily protein, *ROT3*
	orange1.1g012068m.g	AT4G30610	-1.6529	0.000145447	-1.6834	8.36445E-05	-	-	-	-	alpha/beta-Hydrolases, *BRS1*
Cytokinin	orange1.1g020775m.g	AT3G63110	-5.25161	3.2979E-181	-5.70856	5.92047E-28	-	-	-	-	isopentenyltransferase 3, *IPT3*
	orange1.1g001044m.g	AT2G17820	-1.82417	0.002542429	-4.79009	1.08663E-08	-	-	-	-	histidine kinase 1, *HK1*
	orange1.1g008291m.g	AT3G63440	-2.02508	1.27844E-22	-1.13623	1.28232E-06	-	-	-	-	cytokinin oxidase/dehydrogenase 6, *CKX6*
	orange1.1g009956m.g	AT5G21482	-1.84078	9.5059E-07	-2.19491	0.000444096	-	-	-	-	cytokinin oxidase 7, *CKX7*
Gibberellin	orange1.1g039084m.g	AT4G02780	-3.63286	0.042627405	-	-	-	-	-	-	Terpenoid cyclases/Protein prenyltransferases, *GA1*
	orange1.1g018025m.g	AT4G21200	-4.28415	0.006638519	-3.75251	0.043896613	-	-	-	-	gibberellin 2-oxidase 8, *GA2OX8*
	orange1.1g012524m.g	AT1G05160	-3.55185	0.009259011	-3.08975	0.049156991	-	-	-	-	cytochrome P450, family 88, subfamily A, polypeptide 3, *CYP88A3*
	orange1.1g019235m.g	AT3G63010	-1.4383	1.38741E-18	-1.35792	1.94415E-16	-	-	-	-	alpha/beta-Hydrolases, *GID1B*
	orange1.1g034401m.g	AT1G74670	-4.5523	2.3549E-109	-4.17025	3.85587E-25	-	-	-	-	Gibberellin-regulated protein, *GASA6*
