# Supplementary material for: Comparative analysis of the transcriptomes of the calyx abscission zone of sweet orange insights into the huanglongbing-associated fruit abscission
Source: Hortic Res. 2019 Jun 1;6:71. doi: 10.1038/s41438-019-0152-4 (PMC6544638; doi:10.1038/s41438-019-0152-4)
Supplement: Supplementary file 11 — Table S8. JA/ET induced defense response genes [file 41438_2019_152_MOESM11_ESM.pdf]

Table S8. JA/ET induced defense response genes

Citrus Gene ID	Best arabidopsis hit name	Dd/Rd		Dd/Dh		Dh/Rh		Rd/Rh		Gene symbols and descriptions
		Log2FC	P value	Log2FC	P value	Log2FC	P value	Log2FC	P value	
orange1.1g034826m.g	AT1G19610	3.18	6.74E-17	7.73	2.98E-37	-	-	4.55	4.05E-04	*PDF1.4* , plant defensin type 1
orange1.1g032285m.g	AT3G04720	2.05	6.19E-60	4.06	1.18E-168	-1.21	1.44E-13	1.17	2.77E-02	*PR4* (or *HEL*), pathogenesis-related 4
orange1.1g020203m.g	AT5G06860	1.68	9.40E-31	1.01	5.74E-13	-	-	-	-	*PGIP1* , polygalacturonase inhibiting protein
orange1.1g018955m.g	AT3G57270	1.06	7.20E-16	3.13	7.81E-103	-	-	2.2	2.77E-47	*BG1* , beta-1,3-glucanase,
orange1.1g019118m.g	AT5G07010	1.24	1.41E-23	1.74	5.14E-42	-	-	-	-	*ST2A* , sulfotransferase 2A
orange1.1g023970m.g	AT3G01420	6.19	9.59E-66	7.93	6.10E-75	-2.73	4.95E-03	-	-	*DOX1* , alpha-dioxygenase 1
orange1.1g006236m.g	AT2G40140	1.26	1.21E-12	1.65	3.25E-14	-	-	-	-	*CZF1* , zinc finger (CCCH-type) protein
orange1.1g038755m.g	AT1G47510	1.3	6.76E-06	2.27	1.31E-12	-	-	-	-	*5PTASE11* , inositol polyphosphate 5-phosphatase 11
orange1.1g033041m.g	AT3G16720	1.67	8.09E-08	2.23	8.88E-12	-	-	-	-	*ATL2* , ring-H2 finger protein
orange1.1g020013m.g	AT5G64250	1.41	7.00E-29	1.67	1.12E-38	-	-	-	-	Aldolase-type TIM barrel family protein
orange1.1g041268m.g	AT3G10985	1.2	4.33E-18	1.91	1.49E-24	-1.62	6.21E-19	-	-	*SAG20* , senescence associated gene 20
orange1.1g012022m.g	AT1G47128	2.77	6.36E-05	4.53	8.17E-08	-	-	-	-	*RD21A* , granulin repeat cysteine protease
orange1.1g040453m.g	AT2G39210	1.62	6.61E-24	2.13	6.57E-27	-	-	-	-	Major facilitator superfamily protein
orange1.1g026928m.g	AT3G54420	3.05	3.44E-93	5.98	1.74E-276	-	-	2.18	2.15E-28	*CHIV* (or *EP3*), chitinase class IV
orange1.1g044801m.g	AT5G24090	2.47	1.14E-79	3.56	4.20E-109	-	-	-	-	*CHIB1* , chitinase B1
orange1.1g037639m.g	AT4G19810	1.86	4.15E-11	3.68	6.64E-27	-	-	1.27	8.01E-11	*CHIC* , chitinase C
orange1.1g038250m.g	AT5G37490	2.45	5.74E-46	3.82	7.17E-90	-1.03	2.56E-04	-	-	*PUB21* , U-box domain-containing protein 21
orange1.1g014913m.g	AT3G52450	1.93	1.17E-46	2.8	7.93E-75	-1.13	3.16E-05	-	-	*PUB22* , U-box domain-containing protein 22
orange1.1g015513m.g	AT2G35930	2.26	4.09E-40	3.56	2.10E-79	-1.07	8.76E-05	-	-	*PUB23* , U-box domain-containing protein 23
orange1.1g041408m.g	AT3G11840	1.59	6.35E-13	2.65	1.36E-26	-	-	-	-	*PUB24* , U-box domain-containing protein 24
orange1.1g015939m.g	AT3G18710	1.23	2.18E-17	2.38	7.79E-44	-	-	-	-	*PUB29* , U-box domain-containing protein 29
