# Supplementary material for: Comparative analysis of the transcriptomes of the calyx abscission zone of sweet orange insights into the huanglongbing-associated fruit abscission
Source: Hortic Res. 2019 Jun 1;6:71. doi: 10.1038/s41438-019-0152-4 (PMC6544638; doi:10.1038/s41438-019-0152-4)
Supplement: Supplementary file 12 — Table S9. Genes and the primers used in the qRT-PCR [file 41438_2019_152_MOESM12_ESM.pdf]

Table S9. Genes and the primers used in the qRT-PCR

Citrus Gene ID	Gene Symbol	Primers
orange1.1g028566m.g	*ERF1*	TGAGGAAGCTGCTCTGGCTTA / GCCTTCGAGCCACGGATAT
orange1.1g028454m.g	*ERF4*	AAGCAGCACGTGCCTACGA / TTGGGAAGTTTGTCTTGGCTTT
orange1.1g042755m.g	*ERF109*	TCGCAGCGCTCGAAAAC / TCGGTGGAGAAATCGTTGTCT
orange1.1g020953m.g	*EFE*	AAGATGGCCAGTGGATTGATG / TCACCGAGGTTGACAACAATG
orange1.1g017448m.g	*OPR1*	CGCAGCCACATGCAATTTT / CAGCAATGAGAAAGCCACCAT
orange1.1g038593m.g	*JMT*	GCCTGGACAAATACGCAAGAG / CGATTCTCCCACTGCCCTTA
orange1.1g007688m.g	*LOX3*	TGTTAAGGCTGAAAGGGTCCAT / CAGGCGTCCCAAAATTTGA
orange1.1g007464m.g	*JAR1*	ATGCGAAGTACCTGTCTGGAATC / CCCCTGCATAGTGCCTCAAC
orange1.1g019118m.g	*ST2A*	CCCCTGCTTACTTCCAATCCT / TTGGTTATTTGCATAGAGCTTGTATTC
orange1.1g032285m.g	*PR4*	GGTACAGGAGCCCAACAAATTG / CGTCCAAATCTAAGCCTCCATT
orange1.1g023970m.g	*DOX1*	GCATTGGAGCTTTGGAATTATCC / GCCGTCCGTATTTTGAGGAA
orange1.1g020203m.g	*PGIP1*	AGCCTCACTGGCCCCATAC / CGTAGCGTCTTCAGGTTTTTGA
orange1.1g034826m.g	*PDF1.4*	AATGGCGGAAGCAAAAGTGT / CGGCCCCGACCATGT
orange1.1g038250m.g	*PUB21*	ACCGGAACAAGAGGTGCATT / CGCTTCCGCCAAAACACT
orange1.1g041408m.g	*PUB24*	GGCTCTTCCAGGGCAACAA / GCAAAGGCTGTTTGGTGACA
orange1.1g044801m.g	*CHIB1*	GCGACCCGACCAATAATGG / GCCCTGGCAAGTTTTTATTTCA
orange1.1g006236m.g	*CZF1*	GCTTCTGACGATATCTCTGCCTTT / GCTGGGCTCATCAACATCAA
orange1.1g018955m.g	*BG1*	TTTTCCTCTCTCAATACCCCTTCA / CATTAGCAGCCCAAGAAGAAACA
orange1.1g016039m.g	*PAL1*	AGTGCTGAGCAACACAACCAA / TCAGCTGTTTTTCTGGCAGAGA
orange1.1g043449m.g	*OMT1*	TTGAAGGTGGCATCCCATTC / GGATCTTTGCCATGATACTCGTATG
orange1.1g042175m.g	*GGPS1*	TCAGCAATTGGGTCAGAAGGA / GCCTTCACTGCTGATGTCCAT
orange1.1g007379m.g	*NCED3*	AAACGGAGCCAACCCACTT / CCGTCTCCGTCGAAGAAATG
orange1.1g032264m.g	*HVA22E*	TTGTCAGACAGCAAATTAGGCAGTA / CCTTGCCAGTGCCAGTAGGA
orange1.1g006199m.g	*PIN3*	TCCAGAGGACCGACACCAA / CCCGAATCTGGGAGAAGACA
orange1.1g011403m.g	*ROT3*	CACCTTGGCTTCGTGGACTAA / CTTTTTGGTTTCGTCTTGAACGT
orange1.1g020775m.g	*IPT3*	CGCCAGCCATGGATTTATCT / TCGCCTGGAAACCAATAGGT
orange1.1g039084m.g	*GA1*	ACACAGCATGGGTTGCTCTTATT / TGAGGCATGATGGGAATTGA
orange1.1g034401m.g	*GASA6*	GTGGCAAGCTCCGGTCAT / TCGCCGAACACCGATAAGA
orange1.1g018025m.g	*GA2OX8*	GCACAATAATTAGAAAGGGCCTAGTC / TCGTGGAGTGGGATTTGGTT
orange1.1g017322m.g	*JAZ3*	TCCTCTGGATTTATGCCTTTCTCT / GCCATTCCACGCCTTTGAT
